# Minimal Common Oncology Data Elements Genomics Pilot Project: Enhancing Oncology Research Through Electronic Health Record Interoperability at Vanderbilt University Medical Center

**DOI:** 10.1200/CCI.23.00249

**Published:** 2024-06-27

**Authors:** Yanwei Li, Jiarong Ye, Yuxin Huang, Jiayi Wu, Xiaohan Liu, Shun Ahmed, Travis Osterman

**Affiliations:** ^1^Department of Biomedical Informatics, Columbia University Medical Center, New York, NY; ^2^Vanderbilt University, Nashville, TN; ^3^American Muslim Advisory Council, Nashville, TN; ^4^Department of Biomedical Informatics and Medicine, Vanderbilt University Medical Center, Nashville, TN

## Abstract

**PURPOSE:**

The expanding presence of the electronic health record (EHR) underscores the necessity for improved interoperability. To test the interoperability within the field of oncology research, our team at Vanderbilt University Medical Center (VUMC) enabled our Epic-based EHR to be compatible with the Minimal Common Oncology Data Elements (mCODE), which is a Fast Healthcare Interoperability Resources (FHIR)–based consensus data standard created to facilitate the transmission of EHRs for patients with cancer.

**METHODS:**

Our approach used an extract, transform, load tool for converting EHR data from the VUMC Epic Clarity database into mCODE-compatible profiles. We established a sandbox environment on Microsoft Azure for data migration, deployed a FHIR server to handle application programming interface (API) requests, and mapped VUMC data to align with mCODE structures. In addition, we constructed a web application to demonstrate the practical use of mCODE profiles in health care.

**RESULTS:**

We developed an end-to-end pipeline that converted EHR data into mCODE-compliant profiles, as well as a web application that visualizes genomic data and provides cancer risk assessments. Despite the complexities of aligning traditional EHR databases with mCODE standards and the limitations of FHIR APIs in supporting advanced statistical methodologies, this project successfully demonstrates the practical integration of mCODE standards into existing health care infrastructures.

**CONCLUSION:**

This study provides a proof of concept for the interoperability of mCODE within a major health care institution's EHR system, highlighting both the potential and the current limitations of FHIR APIs in supporting complex data analysis for oncology research.

## Teaser

Demonstrating the capability of #mCODE in leveraging structured genomic data @VUMC_Cancer @vumcdbmi

## INTRODUCTION

As the adoption of electronic health records (EHRs) continues to rise, health care providers, medical practitioners, researchers, and patients are experiencing unparalleled levels of data access. The increasing prominence of agile and scalable cloud services for health care expands the potential of EHRs as a cornerstone for data-driven clinical research. Despite their potential, the current landscape of EHRs, especially data on patients with cancer, faces the barrier of limited interoperability. Most EHRs and home health infrastructures today are constructed atop various systems with overlapping functionalities. This has led to the complex challenge of synchronizing data across these systems, resulting in redundancy and inconsistency in data storage.^1,2^ Data integration and standardization in health systems are required to solve the interoperability problem.^3^

**CONTEXT**

**Key Objective**
How does the integration of Epic-based electronic health records (EHRs) with the Minimal Common Oncology Data Elements (mCODE) standard, using the Fast Healthcare Interoperability Resources framework, represent a novel approach to improving data interoperability and transmission in cancer patient care at Vanderbilt University Medical Center?
**Knowledge Generated**
Our project elucidates the transformative potential of integrating mCODE standards with EHR systems, showcasing advancements in data interoperability that can streamline clinical workflows and enhance personalized oncology care. It offers a blueprint for health care institutions on leveraging standardized data to accelerate cancer research and improve patient outcomes through informed, precision medicine approaches.
**Relevance *(F.P.-Y. Li)***
Implementing a common data standard, such as mCODE as demonstrated in this study, can reduce the interoperability barrier when building digital applications to explore institutional EHR data, thereby facilitating the reuse of data for meaningful secondary analyses.*
*Relevance section written by *JCO Clinical Cancer Informatics* Associate Editor Frank P.-Y. Lin, PhD, MBChB, FRACP, FAIDH.

Historically, standards like Health Level 7 (HL7), including HL7v2 and v3, were developed as a solution aiming to promote seamless integration between different health systems. While HL7 has effectively facilitated integration, it struggles with achieving complete data standardization and interoperability given the complexity and lack of commonly used web standards.^4^ To address these issues, the use of Fast Healthcare Interoperability Resources (FHIR), developed by HL7, emerged to leverage advanced web standards and provide a comprehensive solution to interoperability. Focusing on uniform data structures and HTTP-based Representational State Transfer (RESTful) application programming interfaces (APIs) offer a progressive approach to the system thereby making the implementation more accessible.^5^

Building on the FHIR framework, the Minimal Common Oncology Data Elements (mCODE) initiative was launched in 2019 to promote interoperability in the field of oncology research and take advantage of the powerful features of FHIR like API management. Developed by a group of oncologists, informaticians, researchers, and experts in terminologies and standards, mCODE standardizes 23 profiles composed of 90 data elements spanning six primary domains, including patient, laboratory/vital, cancer, genomics, treatment, and outcome.^6^ The mCODE project has the potential to provide a more structured and standardized way to share cancer patient data and facilitate cancer care delivery and research. Furthermore, recent federal mandates dictate that EHR vendors must adopt APIs compliant with FHIR Release 4.0.1 to improve patient data access.^7^ This move, acknowledging the widespread benefits of implementation on FHIR, further demonstrates a promising outlook for the broader adoption of mCODE in the specific field of oncology research.

Nevertheless, the practical application of genomic profiles within mCODE is not widespread among institutions. This limitation is primarily due to the unavailability of computable genomic data within EHRs, thereby presenting obstacles when evaluating the practicality and identifying the advantages of integrating mCODE within the industry.

In this study, we used genomic data from the EHR database of Vanderbilt University Medical Center (VUMC), one of the few distinguished institutions with computable genomic data. We examined the practicality of implementing mCODE genomics profiles using EHR data. To further showcase the potential of mCODE standards for future application integrations, we designed a web application that maximizes the utilization of our institution's genomic data to enhance accessibility. We also provided data visualization to better assist researchers in extracting valuable insights from cancer patient data.

## METHODS

### Data Selection and Preparation

VUMC uses the Epic Clarity database to store EHRs. Our data set included over 12,000 real cancer patient EHRs extracted from the VUMC database that contained patient demographic information and their genomic test results. In total, we collected data on 1,055 unique gene alterations, 45,279 amino acid changes, and 10,417 cancer diagnoses. To decrease complications associated with the data migration process and to avoid contaminating the VUMC EHR database, we constructed a sandbox environment in Azure to store the VUMC Clarity patient data. We will refer to this created database as the Temporary Database for simplicity, but it is important to note that it is the same structure but not the same EHR database used by VUMC. The Temporary Database in Azure was architected to minimize any impact on the primary Production reporting database of VUMC. No transformation occurred at this stage.

### Deploying FHIR Server

As mCODE extends from FHIR R4 standards and uses a similar set of API calls, we deployed the open-source FHIR server developed by Microsoft on Azure to serve as our FHIR server.^8^ An Azure SQL database that follows the entity attribute value (EAV) model was automatically deployed during the process. All interactions with this database, as well as any internal structural alterations, were managed by the FHIR server, and there is no direct access available to interfere with it. The Azure FHIR server supports all API calls stated within the FHIR R4 IG. After mapping the patient data from the Temporary Database to the new mCODE standard, we loaded all mCODE profiles through this Azure FHIR server and connected our web application to the server to query data stored in the Azure database.

### Data Mapping

This project focused on transforming the EHRs in Temporary Database to implement the Cancer Patient profile and the Genomics profile defined in the mCODE standard.

**FIG 1.**
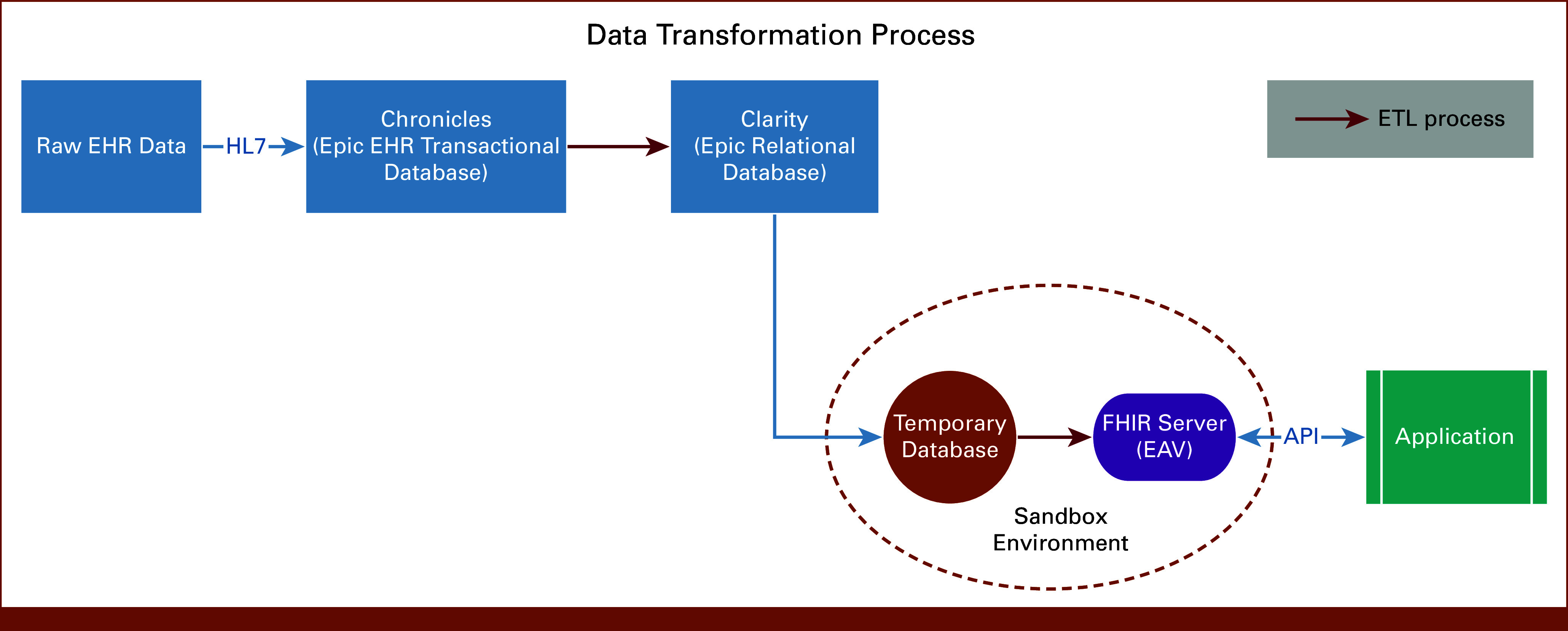
Data workflow: progression of data through various stages from transformation to application. API, application programming interface; EAV, entity attribute value; EHR, electronic health record; ETL, extract, transform, load; HL7, Health Level 7.

The progression of data from collection to application use is depicted in Figure 1. Genomic results, initially communicated through HL7 messages, are ingested by Epic's Chronicles database, a transactional database that serves as a digital ledger for storing and managing patient transactions. From Chronicles, the data were transferred to the Clarity database, where it is structured into tables and columns suitable for analysis. In the real-world production process, data transformation into compatible mCODE profiles would happen directly if the Chronicle database contained the necessary FHIR profiles for full mCODE implementation. However, to avoid any possible contamination of the production database, we created a sandbox environment that only extracted a subset of the Clarity database to test the validity of mCODE implementation. Thus, no data transformation was made when moving the patient data from Clarity database to the Temporary Database.

An extract, transform, load (ETL) tool encompassing three pivotal steps was used to transition the raw Clarity data in the Temporary Database into the structured mCODE format: (1) extracting the EHRs from the Temporary Database; (2) transforming extracted EHRs into mCODE profiles; and (3) loading the transformed mCODE profiles to the Azure FHIR server which automatically validates the profile and stores them into EAV format in its accompanied database. Subsequently, a clinical application is connected to the FHIR server to demonstrate the transformation step is successfully implemented.

In particular, the transformation step required mapping the selected data entries of the Clarity EHRs to their corresponding mCODE data fields for the Cancer Patient profile and Genomic profiles, which included four subprofiles: Genomics Report Profile, Genomic Variant Profile, Genomic Region Studied Profile, and Genomic Specimen Profile. To implement those profiles, we located the required data fields within the Clarity database according to mCODE standards and extracted these to a separate relational database system. Next, we mapped the selected data entries to their corresponding mCODE fields and created the various mCODE profiles. If no matching data could be found to fill in an mCODE Must Support data field, a data absent reason was loaded as a placeholder. For important patient data from the Temporary Database that had no specific location to store in an mCODE profile, we included such information as extensions of their related data field. The last step for data mapping used the bulk import feature of the Azure FHIR server to accomplish loading constructed data.

### Website

**FIG 2.**
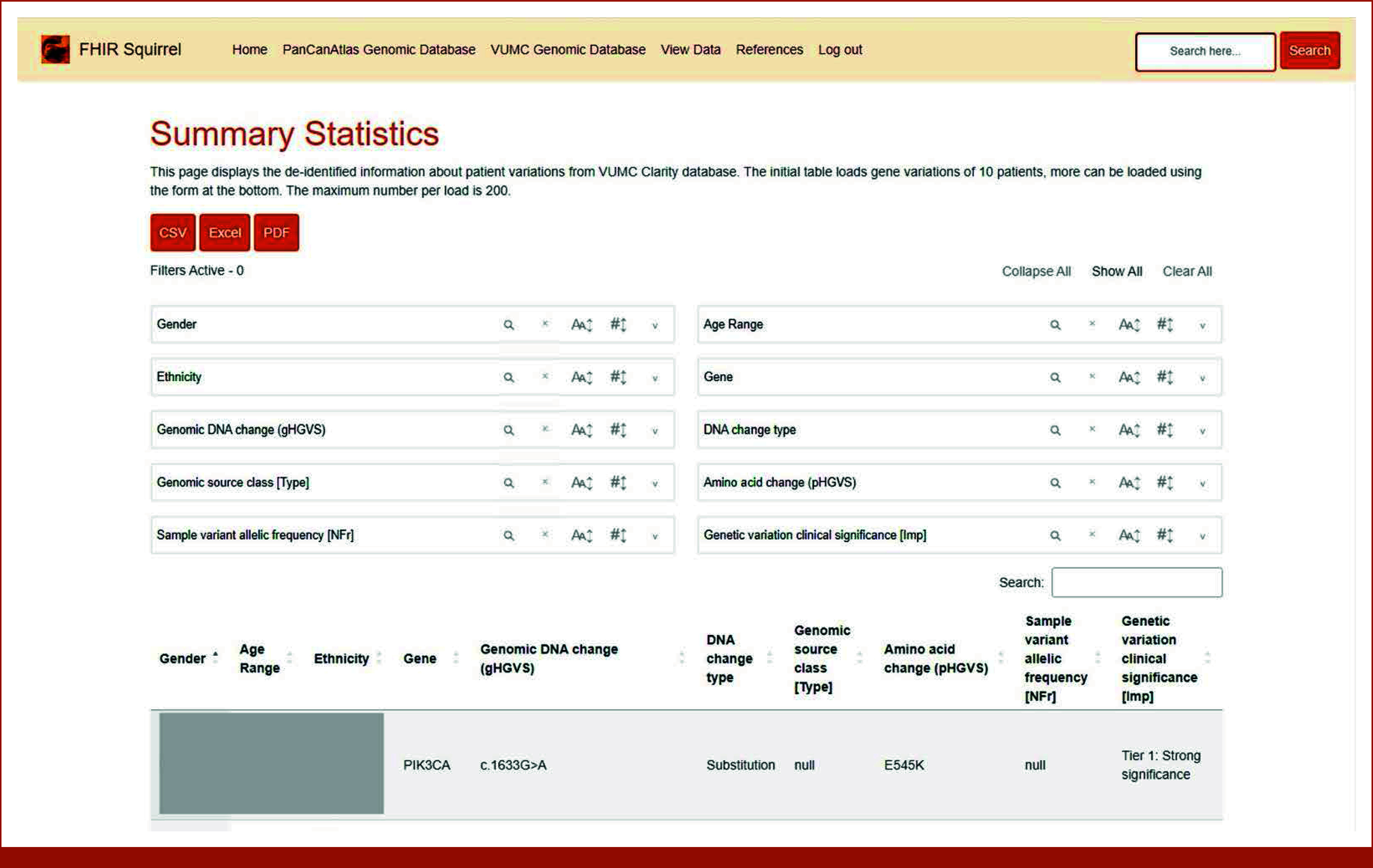
Summary statistics page providing a secure interface for viewing and downloading deidentified patient genetic information, with the option of filtering on the basis of demographic and genomic criteria. FHIR, Fast Healthcare Interoperability Resources; mCODE, Minimal Common Oncology Data Elements; VUMC, Vanderbilt University Medical Center.

**FIG 3.**
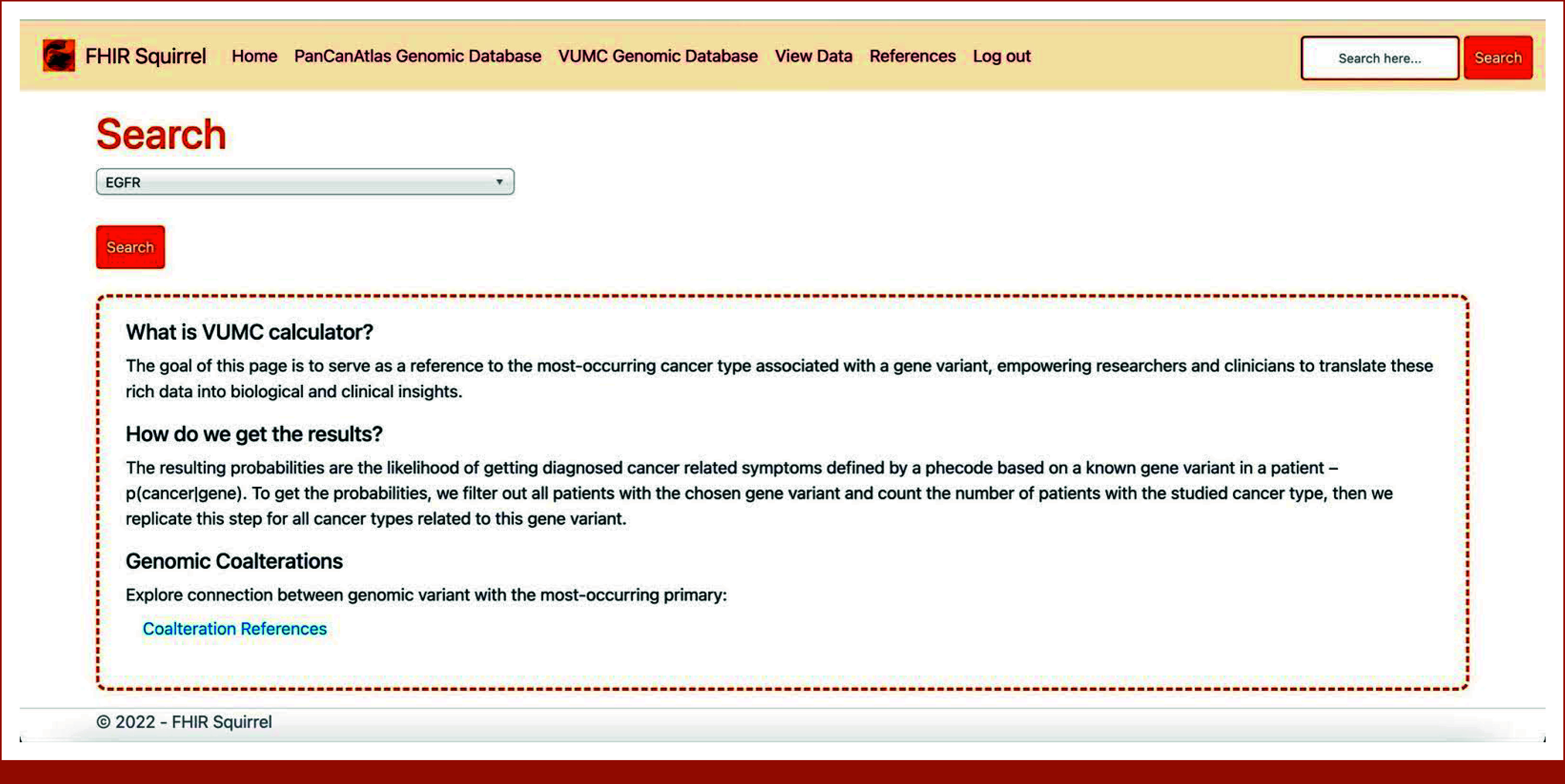
VUMC cancer incidence calculator interface enabling researchers to identify the most frequently occurring cancer type associated with specific gene variants. FHIR, Fast Healthcare Interoperability Resources; VUMC, Vanderbilt University Medical Center.

**FIG 4.**
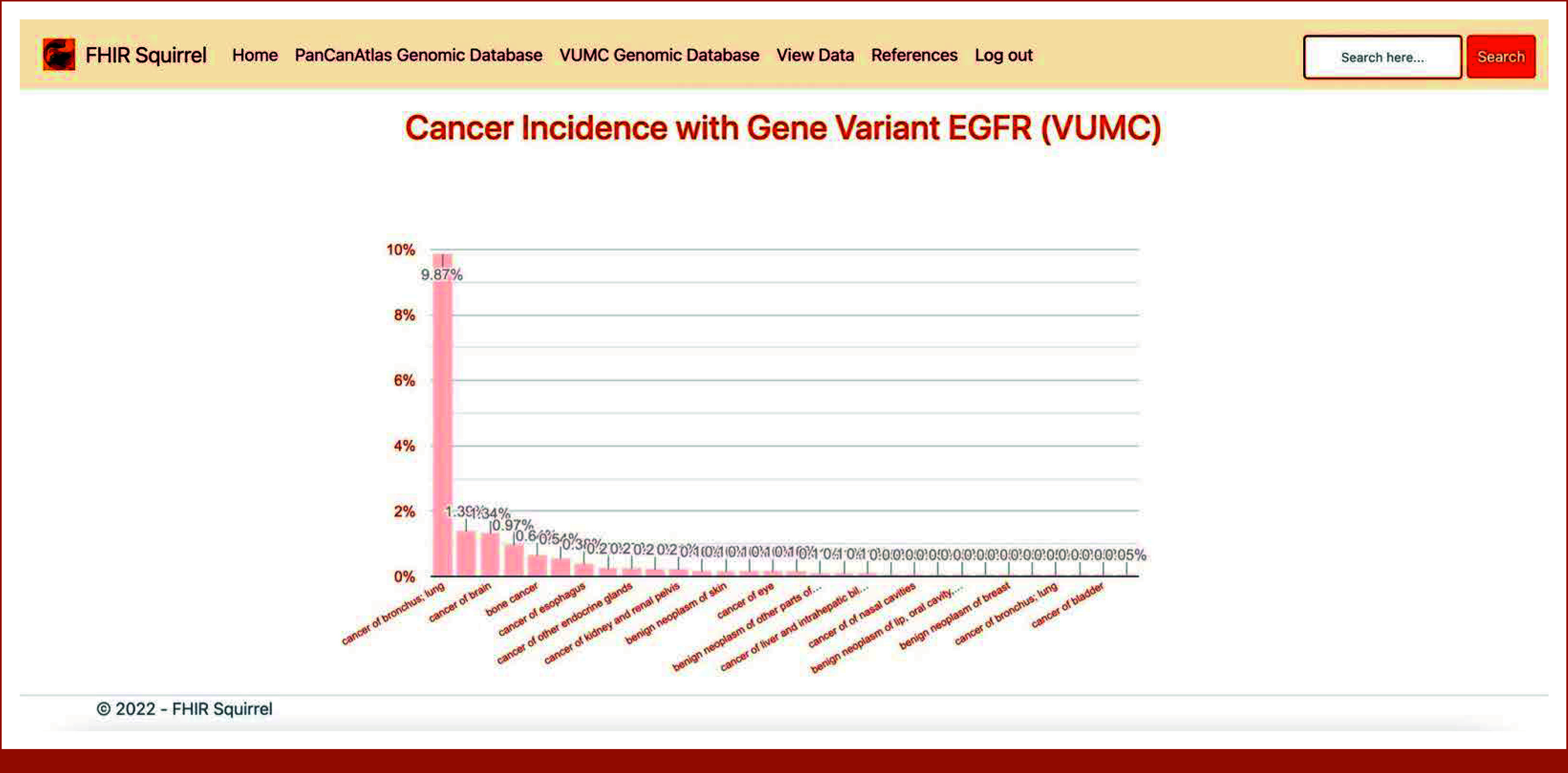
Incidence rates of various cancers associated with the EGFR gene variant, as calculated from gene and Phecode diagnosis data within the Temporary Database. FHIR, Fast Healthcare Interoperability Resources; VUMC, Vanderbilt University Medical Center.

**FIG A1.**
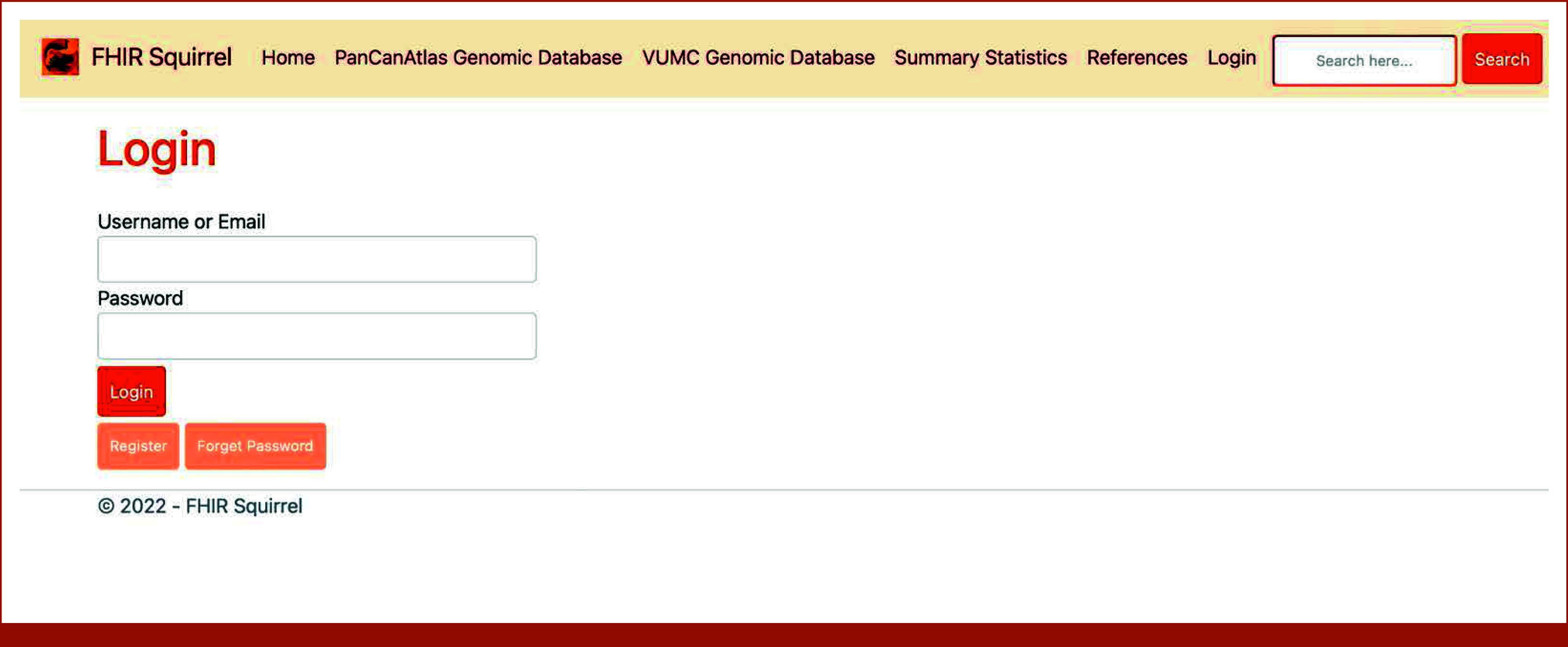
FHIR Squirrel login page where users are prompted to enter their username or e-mail address along with their password, enabling access to the mCODE genomics pilot project. FHIR, Fast Healthcare Interoperability Resources; VUMC, Vanderbilt University Medical Center.

**FIG A2.**
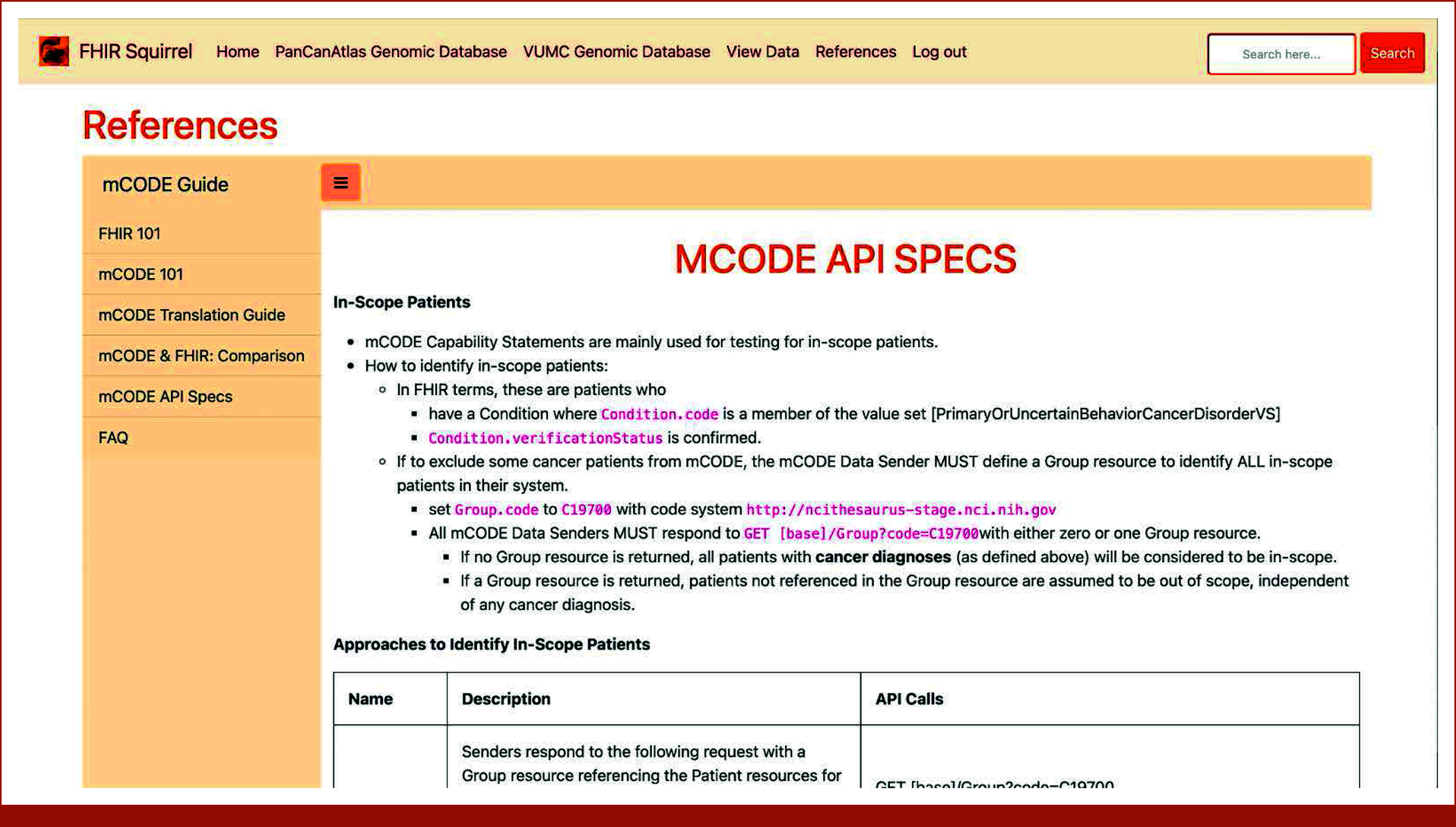
FHIR Squirrel reference page displaying documentation for FHIR and mCODE-related questions. API, application programming interface; FHIR, Fast Healthcare Interoperability Resources; mCODE, Minimal Common Oncology Data Elements; VUMC, Vanderbilt University Medical Center.

After the data migration process, we started designing the front-end side of the web application. The final deliverables of the web application consisted of the following pages: (1) a home page that visualizes all the Phecode diagnoses and genes present in the Temporary Database (Appendix Fig A1); (2) a summary page for requesting and downloading patient data for VUMC users (Fig 2); and (3) two pages for evaluating the risk of cancer incidence based on identified genomic variations, one calculated based on the Temporary Database and the other based on the dataset acquired from The Cancer Genome Atlas (TCGA) Program^9,10^ (Figs 3 and 4). A reference page was also included that presents all documentation related to this study, including a brief introduction and in-depth analysis of both FHIR and mCODE standards (Appendix Fig A2).

The primary objective for this web application was twofold. First, the summary page offers users the ability to view and download deidentified patient information. It provides the functionality to sort the patient database by criteria such as race, sex, age, and genomic alteration. Each row corresponds to a single gene variation of a patient, with details like DNA change, DNA change type, genomic source class, amino change, allelic frequency, and clinical significance, allowing for the exploration of specific relationships between certain genomic mutations and demographics. This feature can only be accessed by users connected to the VUMC network for security reasons.

**FIG 5.**
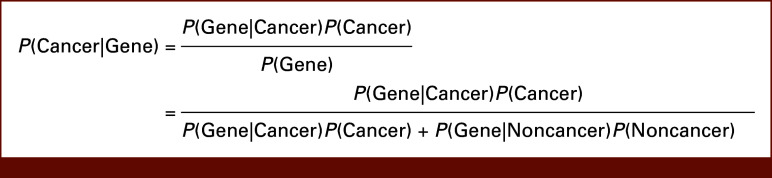
Conditional probability of having one of the 33 cancers profiled by TCGA program given the presence of a known gene variant, using Bayes' theorem. FHIR, Fast Healthcare Interoperability Resources; TCGA, The Cancer Genome Atlas; VUMC, Vanderbilt University Medical Center.

Second, with the extensive data in the Temporary Database and TCGA program, we computed the likelihood of developing one of the 33 cancer types profiled by the TCGA program, given the presence of a known gene variant. This functionality enables a more nuanced understanding of the genomic underpinnings of cancer, paving the way for targeted diagnostics and personalized treatment strategies. The underlying logic was based on Bayes' Theorem, commonly used to calculate the probability of an event occurring on the basis of previous conditions. In the context of our research, we applied Bayes' Theorem to determine the conditional probability of being diagnosed with one of the cancer types on the basis of the presence of a specific gene variant. The equation, as applied in our analysis, is shown in Figure 5.

## RESULTS

### Data Migration

To facilitate the transfer of VUMC EHR data from VUMC Epic Clarity database into mCODE profiles, we used the developed ETL tool that extracted data from Clarity, compiled corresponding data into mCODE profiles, and uploaded in bulk the constructed profiles to the Azure FHIR server. Data from 12,000 patients with over 80,000 variant profiles were loaded onto the Azure FHIR server. Concurrently, each profile underwent validation at the time of being loaded into the FHIR server. The migration process took roughly a day to complete; during the process, a bottleneck of the migration step was detected, which was the step of making external API calls to the FHIR server from the ETL tool. To circumvent this issue, a multithread processing feature in Spring Boot was used that significantly improved the speed of uploading profiles to the Azure FHIR server by allowing simultaneous and nonblocking posting of constructed profiles to the server.

### Web Application

**FIG 6.**
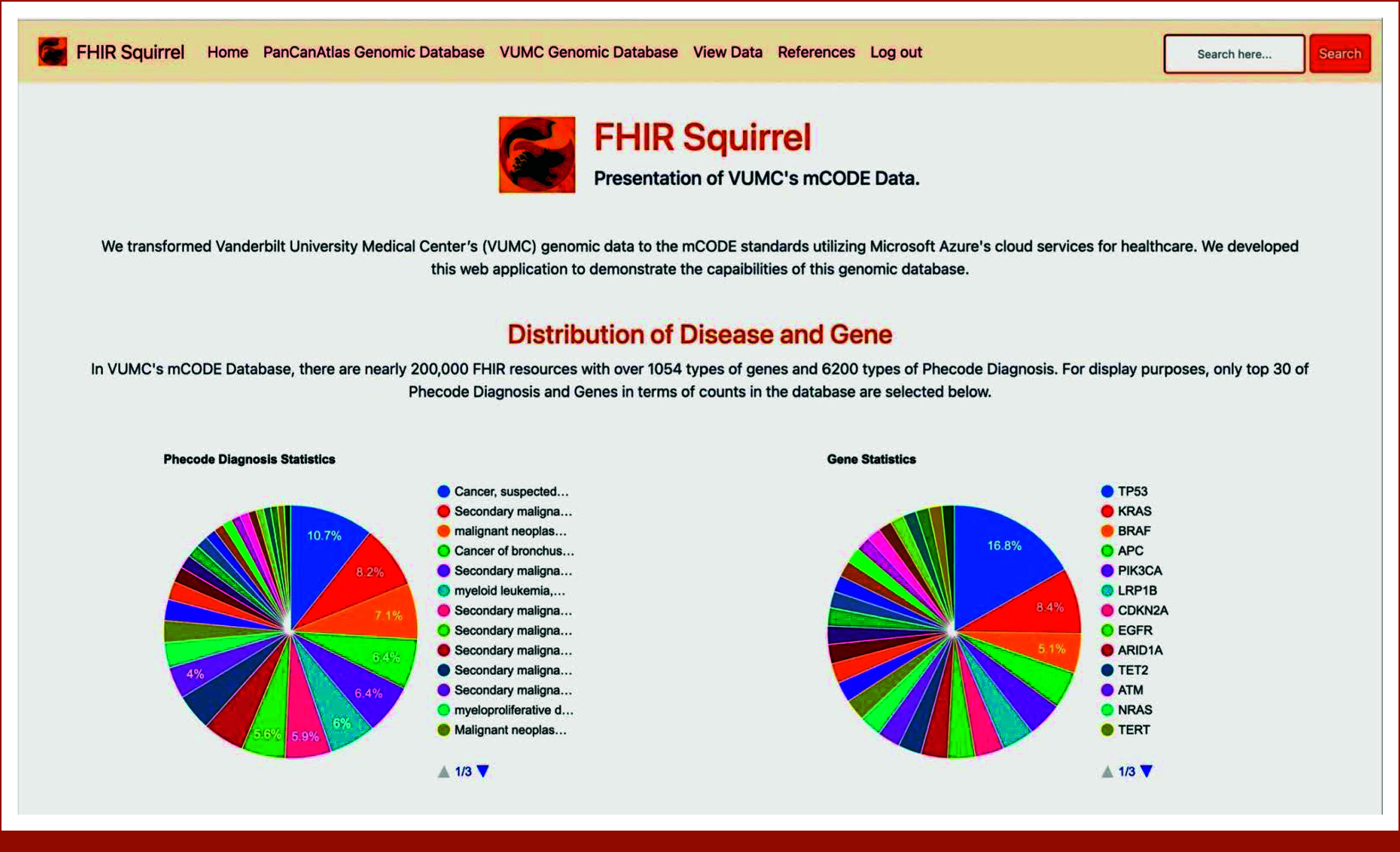
FHIR Squirrel home page providing interactive pie charts, visualizing gene and Phecode diagnosis distribution in Temporary Database. FHIR, Fast Healthcare Interoperability Resources; mCODE, Minimal Common Oncology Data Elements; VUMC, Vanderbilt University Medical Center.

The finished website application has five main functionalities implemented on five separated pages. An account registered with a VUMC email is required to access any content on the website other than login, ensuring data security and preventing patient data breach (Appendix Fig A1). The home page displays the distribution of cancers and genes within the database as two colored pie charts, shown in Figure 6. The chart on the left displays the most common Phecode diagnosis and their respective percentage within our Azure database; the chart on the right shows the most common gene variations and their percentages. It is worth noting that since there is a large number of diseases and gene variations in the database, only the top 30 are shown on the home page for more concise visualization.

The cancer incidence calculator is presented on two separate web pages: one uses the Temporary Database and the other is based on data from the TCGA program. Figures 3 and 4 provide illustrations of the calculator's application using the Temporary Database.

The summary page in Figure 2 displays a summary table of genomic profiles with a focus on gene variation. Before loading the page, an alert message will appear that asks whether the user is on the VUMC virtual net. Users who are not connected to the virtual network will be restricted to view only the patient data to protect patient privacy. The summary page first presents a variation of 10 patients in the database; users have the choice to load a custom number of patients following the initial 10 patients up to 200 patients. A user can choose to load all patients, but this option will trigger a warning message about possible waiting times.

## DISCUSSION

A key decision made during the project was to avoid directly interacting with the Clarity database at VUMC because of the additional oversight and complexity that accompany production environment modifications. For this reason, a sandbox environment was established, including the Temporary Database, which allowed for developmental flexibility and testing without the risk of compromising the stability of the primary health record system.

The central challenge in the data mapping and transformation process is the necessity to map data from the Epic Clarity relational database to the EAV-styled mCODE format. This task becomes particularly formidable because of the absence of FHIR resources at VUMC's production environment and the widespread lack of implementation in many institutions across the country. The unavailability of FHIR resources complicates the exploration of the path for transforming Clarity data into mCODE profiles. Within this context, challenges arise, including disparities in naming conventions, variations in code systems, and the need to transform table-based data structures into profile-based formats. These factors collectively contribute to the complexity of the data migration process.

One of the first challenges faced during the data extraction stage was the discrepancy in naming conventions and code systems. The same data field may be named differently in the Clarity database and in the mCODE format, which required a careful search within the official documentation of Epic Clarity databases and the mCODE implementation guide to find matches. Medical professionals were also consulted to assist in connecting the lines between the data.

Another challenge encountered while transforming and compiling patient data into mCODE profiles was assembling data scattered across various tables into the six proposed mCODE profiles. The structure of various mCODE profiles and their dependency relationships had to be fully understood to ensure the data were placed correctly. Although all must-support elements in mCODE profiles can be implemented, as data absent reason elements can be placed in fields that lack corresponding information in the EHR database, there remain many parts in genomics profiles that the Clarity database only provide empty value, causing some profiles to be only complete in structure but lacking important contents.

The integration of FHIR aimed to promote data interoperability and advance oncology research. However, our experience highlighted that stemming from the design of FHIR, FHIR tends to be better suited for managing individual patient-level data rather than population-level data. As a result, employing FHIR for analytics presented challenges, especially in contexts requiring comprehensive data aggregation and analysis at the population level.

To generalize this project for other institutions, it is essential to recognize that while the Epic Clarity database maintains a high level of structural uniformity across sites, minor schema variations can occur with different Epic versions. Additionally, some institutions may opt out of extracting certain tables for their FHIR resource construction, though these variances are minor. Although our project targets VUMC Clarity data, the transformation process is designed to be adaptable. Besides, direct transformation from Clarity is not the sole method; institutions with comprehensive FHIR profiles for mCODE can directly apply mCODE transformations to these profiles.

One priority when working with EHR and patient data is to ensure data safety and protect patient privacy. For these purposes, the web application has multiple layers of protection implemented. However, not all fields require multiple layers of authorization. Most data visualization pages do not require users to be on the VUMC virtual net. The homepage pie charts draw from precalculated statistics stored in static Azure FHIR database tables to lower querying overhead. Other features like the PanCanAtlas and VUMC genomic calculators also bypass the private server for data retrieval. Although most planned functionalities were accomplished, several problems were encountered that stopped the team from implementing more advanced features. The current search function only scans static content for titles and links without text highlighting capabilities. Additionally, owing to Azure FHIR API constraints, data loading on the summary page is capped at 200 rows to prevent delays, and the FHIR server's limit of 1,000 profiles necessitates multiple API calls for comprehensive data, leading to significant wait times.

In conclusion, our project has illuminated several key insights regarding the application of mCODE. First, by migrating VUMC's EHR data into an Azure FHIR database, we have demonstrated the potential of cloud-based EHR data management. The transition to a cloud environment not only provides secure storage but also allows for the integration of vital authentication services.

Furthermore, this project has shown the possibility of building mCODE genomic profiles on existing real-world EHRs. However, this integration necessitates consideration of database structures to align with the specifics of mCODE genomic data. Presently, incongruities exist between the mCODE standard and prevailing EHR data standards, mandating a manual data mapping process to transition between those two standards.

Moreover, our efforts culminated in developing a web application utilizing mCODE FHIR APIs. Yet, we encountered limitations in statistical methodologies, with many FHIR APIs lacking support for such analytical techniques. This underscores the need for updates and enhancements to the existing FHIR API standard, particularly focusing on bolstering support for statistical methodologies, and consequently optimizing the application of FHIR data for medical research purposes.

Despite the mentioned challenges, our project still affirms the applicability of the mCODE standard in constructing applications centered around genomics and oncology data. This affirmation holds great promise, particularly if future updates enhance mCODE and FHIR standard databases to better support data retrieval for statistical analysis, further amplifying its potential impact in the domain of medical research and care.
